# Phage-antibiotic combinations against *Klebsiella pneumoniae*: impact of methodological approaches on effect evaluation

**DOI:** 10.3389/fmicb.2025.1530819

**Published:** 2025-03-12

**Authors:** Roman B. Gorodnichev, Anastasiia O. Krivulia, Maria A. Kornienko, Narina K. Abdraimova, Maja V. Malakhova, Marina V. Zaychikova, Dmitry A. Bespiatykh, Valentin A. Manuvera, Egor A. Shitikov

**Affiliations:** ^1^Department of Biomedicine and Genomics, Lopukhin Federal Research and Clinical Center of Physical-Chemical Medicine of Federal Medical Biological Agency, Moscow, Russia; ^2^Moscow Center for Advanced Studies, Moscow, Russia

**Keywords:** phage, antibiotic, capsule depolymerase, synergy, antagonism, *Klebsiella pneumoniae*

## Abstract

**Background:**

The combined use of bacteriophages and antibiotics represents a promising strategy for combating multidrug-resistant bacterial pathogens. However, the lack of uniformity in methods for assessing combination effects and experimental protocols has resulted in inconsistent findings across studies. This study aimed to evaluate the effects of interactions between phages and antibiotics on *Klebsiella pneumoniae* strains using various statistical approaches to formalize combination effects.

**Methods:**

Effects were assessed for four antibiotics from distinct classes (gentamicin, levofloxacin, meropenem, chloramphenicol), three phages from different genera (Dlv622, Seu621, FRZ284), and a depolymerase (Dep622) on three *K. pneumoniae* strains of the KL23 capsule type. Antibiotics were used at C_max_ concentrations, and phages at sublethal levels. A modified *t*-test, Bliss independence model, two-way ANOVA, and checkerboard assay were employed to evaluate the results.

**Results:**

Among 48 combinations, 33 effects were statistically significant, including 26 cases of synergy and 7 of antagonism. All statistical methods showed consistency in identifying effects; however, the *t*-test and Bliss method detected a greater number of effects. The strongest synergy was observed with levofloxacin in combination with Seu621 or Dep622 across all bacterial strains. Checkerboard assays confirmed synergy in selected cases but indicated that combined effects could vary with antimicrobial concentrations.

**Conclusion:**

The choice of analytical method substantially impacts the detection of phage-antibiotic effects. The *t*-test and Bliss method, due to their simplicity and sensitivity, may be optimal for clinical application, while two-way ANOVA for confirming strong interactions. These results emphasize the need to consider interaction characteristics when designing therapeutic strategies.

## 1 Introduction

*Klebsiella pneumoniae* is both a natural component of human microbiota, colonizing the gastrointestinal tract and nasopharynx in healthy individuals (Chang et al., 2021), and a dangerous pathogen, responsible for approximately 11–15% of hospital-acquired pneumonia cases and up to 8% of all healthcare-associated infections (Chen et al., 2023; Grosjean et al., 2024; Rahmat Ullah et al., 2024).

Infections caused by *K. pneumoniae* represent a significant concern in healthcare settings due to the increasing prevalence of antibiotic-resistant strains. In Europe, over one third of *K. pneumoniae* isolates demonstrate resistance to at least one major antimicrobial drug class, including fluoroquinolones, third-generation cephalosporins, aminoglycosides, or carbapenems (European Centre for Disease Prevention and Control, 2022). These infections are responsible for an estimated 650,000 deaths annually worldwide (Murray et al., 2022). In light of this significant public health concern, the World Health Organization (WHO) has identified *K. pneumoniae* as a high-priority pathogen in its 2024 Bacterial Priority Pathogens List (World Health Organization, 2024).

One promising alternative for combating infections caused by antibiotic-resistant strains is bacteriophage (phage) therapy and its derivatives, including polysaccharide depolymerases and endolysins (Danis-Wlodarczyk et al., 2021; Durbas and Machnik, 2022). Bacteriophages—viruses that specifically infect and lyse bacteria—have demonstrated promising results in treatment applications (Pirnay et al., 2024). Although clinical data on recombinant phage proteins in humans are still unavailable, their efficacy has been convincingly demonstrated in animal models (Volozhantsev et al., 2020; Gorodnichev et al., 2021a; Hua et al., 2022; Li et al., 2024).

Phage therapy is commonly implemented under Article 37 of the Declaration of Helsinki, providing “compassionate care” when conventional antimicrobial therapies fail. However, standard treatment protocols generally do not recommend antibiotic withdrawal, meaning that phage therapy is often administered concurrently with antibiotics. This raises important questions about the potential interactions between these antimicrobial agents.

The combined use of phages and antibiotics can yield varying outcomes: synergy, additive effect, or antagonism. Synergy occurs when the bactericidal action of antimicrobial agents together is greater than the sum of their individual effects. Additive effects indicate an impact equal to the sum of the individual effects, while antagonism describes an interaction where the action of one agent inhibits the effect of the other (Liu et al., 2020).

In therapeutic contexts, antagonistic interactions between phages and antibiotics, which can reduce overall treatment efficacy, are considered particularly critical—even more so than synergy among antimicrobial agents. Reflecting this, several phage therapy centers have incorporated *in vitro* tests for detecting combined effects of phages and antibiotics as part of the standard phage selection process (Onallah et al., 2023).

To date, no cases of antagonism between phages and antibiotics targeting *K. pneumoniae* have been documented. Instead, both *in vitro* (Bedi et al., 2009; Verma et al., 2010; Qurat-ul-Ain et al., 2021; Han et al., 2022; Xu et al., 2022; Lew et al., 2023) and *in vivo* (Chhibber et al., 2008; Pacios et al., 2021; Wang et al., 2021) studies have demonstrated high efficacy of these agents when used together. However, differences in bacterial strains, phages, antibiotics, and methodologies often complicate comparisons across studies.

The aim of this work was to compare various *in vitro* methods for assessing combination effects of antibiotics and phages. Using three strains of *K. pneumoniae*, we evaluated the individual and combined actions of four antibiotics (gentamicin, levofloxacin, meropenem, and chloramphenicol), three phages (Dlv622, Seu621, and FRZ284), and a depolymerase (Dep622). To quantify combination effects, we applied the most commonly used methods for such studies: the *t*-test, Bliss independence model, two-way ANOVA, and checkerboard assay.

## 2 Materials and methods

### 2.1 Bacterial strains and characteristics

This study utilized *K. pneumoniae* strains 9faize, G4-17, and B536-17-2, isolated from patients between 2018 and 2023. Prior to use, the strains were propagated in lysogeny broth (LB, HiMedia, India) at 37°C. Capsule typing of *K. pneumoniae* was conducted via sequencing of the *wzi* gene (Brisse et al., 2013), and sequence typing followed the standard MLST protocol (Diancourt et al., 2005). Antibiotic susceptibility was determined using the broth microdilution method according to Clinical & Laboratory Standards Institute (CLSI) guidelines (CLSI, 2015). The antibiotics used included gentamicin (GEN), levofloxacin (LVX), meropenem (MEM), and chloramphenicol (CMP) (HiMedia, India).

### 2.2 Bacteriophages

Three previously characterized *K. pneumoniae* phages Dlv622, Seu621, and FRZ284 (GenBank accessions MT939252, MT939253, and MZ602148) were included in the study, classified under the genera *Drulisvirus*, *Mydovirus*, and *Jiaodavirus*, respectively (Gorodnichev et al., 2021a; b). Phage titers and efficiency of plating (EOP) were determined using the spot test and double-layer agar methods (Kutter, 2009; Mazzocco et al., 2009).

Lytic activity of the phages was assessed using Appelman’s titration method (Burrowes et al., 2019). For this, phage suspensions were serially diluted 10-fold in LB broth. A total of 190 μL of each dilution was added to microplate wells, followed by inoculation with 10 μL of bacterial culture in the logarithmic growth phase at a concentration of 10^5^ CFU/well. The plates were incubated overnight at 37°C, and lytic activity was determined as the lowest dilution that inhibited bacterial growth.

### 2.3 Polysaccharide depolymerase

The recombinant polysaccharide depolymerase Dep622, derived from phage Dlv622, was previously obtained and characterized (Gorodnichev et al., 2021a). The minimum halo-forming concentration (MHF) of depolymerase was assessed using a method similar to phage titration. For the assay, 7 mL of molten, semi-solid LB agar (0.7%) was combined with 100 μL of an overnight culture of the test strain, vortexed, and spread evenly across the surface of a solidified LB agar plate. After solidification, 5 μL of serially diluted recombinant depolymerase (prepared in a 2-fold LB dilution series) was applied to the agar surface using a template. The plate was incubated at 37°C for 18 h, and translucent spots were observed on the bacterial lawn. The MHF was defined as the lowest concentration that produced visible spots.

### 2.4 Individual and combined effects of antimicrobial agents on planktonic bacterial cells

The individual and combined effects of antibiotics, phages, and depolymerase were assessed using time-kill curves. Antimicrobial agents were added individually or in combination to the wells of a 96-well plate. Antibiotics were applied at concentrations equivalent to their maximum serum concentration (C_max_) (Table 1). Phage lysates were used at a sublethal concentration based on Appelman’s titration (10^9^ PFU/well), while recombinant depolymerase at 100 MHF. A positive bacterial growth control included no antimicrobial agents, while control wells for individual agents contained only the antibiotic, phage, or depolymerase. Wells were inoculated with 10 μL of each strain in the exponential growth phase (OD_600_ = 0.3), diluted in LB to achieve 10^5^ CFU/well. The final volume in each well was 200 μL. Optical density (OD) was measured every hour for 20 h at 37°C using a FlexA200 microplate reader (Allsheng, China) at 600 nm.

**Table 1:** MIC breakpoints for *K. pneumoniae* and maximum concentration of antibiotic in serum.

	CLSI (μg/mL)			C_max_ (μg/mL)
	≤S	I	R≥	
Gentamicin	4	8	16	16 (Gonçalves-Pereira et al., 2010)
Levofloxacin	2	4	8	6 (Wagenlehner et al., 2006)
Meropenem	1	2	4	28 (Thalhammer et al., 1998)
Chloramphenicol	8	16	32	25 (Wenk et al., 1984)

The individual effects of antibacterial agents on planktonic cells were evaluated by calculating the percent reduction in optical density, according to the formula: (1 - ((OD of treated - OD_K−_)/(OD of untreated - OD_K−_))) × 100%. The endpoint and area under the curve (AUC) data were used for the calculation. The AUC was determined using the trapezoidal rule.

Statistical analyses, including *t*-tests, two-way ANOVA, and Bliss analysis, were used to evaluate combined effects.

Based on endpoint OD_600_ measurements, a *t*-test was utilized to assess differences between OD_600_ values of the antimicrobial combinations and their individual actions. Antagonism was indicated when the combination’s OD_600_ was significantly higher than that of the most effective agent alone, or when the OD_600_ of the combination did not differ from the most effective agent, while the second agent showed a significant effect compared to the control. Synergy was defined when the combination’s OD_600_ was significantly lower than the most effective agent, while the second agent’s OD_600_ was not different from the control. When the OD_600_ of the combination was significantly lower than that of the most effective agent, and both individual agents also showed a significant reduction compared to the control, this phenomenon was termed “tentative synergy.”

Bliss analysis followed the approach of Eller et al. (2021). From endpoint OD_600_ data, a synergy coefficient (*S*) and *p*-value were calculated, where *S* > 0 indicated synergy and *S* < 0 indicated antagonism. The significance of the *S* value was assessed using a one-sample *t*-test with a hypothesized mean of zero, accounting for individual propagated errors.

Two-way ANOVA was conducted following Gordillo Altamirano et al. (2022) for both endpoint OD_600_ and the area under the curve, calculated using the trapezoidal rule. Data normality was verified using the Shapiro–Wilk test, and multiple comparisons were corrected with Tukey’s test. Interaction plot slopes were used to interpret types of combined effects.

Statistical analyses were performed using GraphPad Prism 8. Any effects not meeting the criteria for statistical significance (*p* < 0.05) were considered additive.

### 2.5 Checkerboard assay

A modified checkerboard assay (Nikolic et al., 2022) was performed for specific combinations of antimicrobial agents. Antibiotic concentrations ranged from 2 to 128 μg/mL across columns, while phage titers varied from 10^3^ to 10^9^ PFU/well across rows. The first row contained only phages, and the last row included only antibiotics. The bottom-left well served as a positive control with no antimicrobial agents. Each well was inoculated with 10 μL of bacterial culture in the exponential growth phase (OD_600_ = 0.3), diluted in LB to a final concentration of 10^5^ CFU/well.

The plates were incubated for 20 h at 37°C, and OD was measured hourly at 600 nm using a FlexA200 microplate reader (Allsheng, China). Each experiment was conducted in triplicate, with results averaged.

To evaluate the effect of phage-antibiotic interactions, the Fractional Inhibitory Concentration Index (FICI) was calculated:


$$\mathrm{FICI}=\frac{{\mathrm{MIC}}_{\mathrm{of phage in combination}}}{{\mathrm{MIC}}_{\mathrm{of phage alone}}}+\frac{{\mathrm{MIC}}_{\mathrm{of antibiotic in combination}}}{{\mathrm{MIC}}_{\mathrm{of antibiotic alone}}}$$

FICI values were interpreted as follows: FICI <0.5 indicated synergy, FICI between 0.5 and 1 indicated an additive effect, 1 < FICI <2 was considered indifferent, and FICI >2 indicated antagonism. Data visualization was performed using ComplexHeatmap v2.16.0 for R v4.3.0 and SynergyFinder v3.0 (Gu et al., 2016; Ianevski et al., 2022).

## 3 Results

### 3.1 Susceptibility of *Klebsiella pneumoniae* strains to antibiotics, phages, and depolymerase

The *K. pneumoniae* strains were isolated from clinical samples between 2018 and 2023, belonged to capsule type KL23 and clinically relevant sequence types (Table 2). All strains were resistant to the antibiotics tested, according to CLSI standards.

**Table 2:** Characterization of sensitivity of *K. pneumoniae* strains to antimicrobial agents.

Strain	9faiz	G4-17	B536-17-2
Year of isolation	2019	2023	2018
Capsular type	KL23	KL23	KL23
Sequence type	39	39	1869
Antibiotic resistance			
Gentamicin MIC	>128 μg/mL (R)	>128 μg/mL (R)	>128 μg/mL (R)
Levofloxacin MIC	128 μg/mL (R)	64 μg/mL (R)	128 μg/mL (R)
Meropenem MIC	32 μg/mL (R)	>128 μg/mL (R)	64 μg/mL (R)
Chloramphenicol MIC	>128 μg/mL (R)	>128 μg/mL (R)	>128 μg/mL (R)
Phage susceptibility			
Dlv622 EOP	0.01	0.001	1
Seu621 EOP	0.01	0.1	1
FRZ284 EOP	Lysis from without	Lysis from without	Lysis from without
Dlv622 Appelman titer	>10^0^	>10^0^	>10^0^
Seu621 Appelman titer	>10^0^	>10^0^	>10^0^
FRZ284 Appelman titer	>10^0^	>10^0^	>10^0^
Depolymeraze susceptibility			
Dep622 MHF	0.68 μg/mL	1.37 μg/mL	0.68 μg/mL

Three phages were tested: two capsule-specific phages (Dlv622 and Seu621) and one broad-host-range phage FRZ284 that was not capsule-dependent (Gorodnichev et al., 2021a; b). Phages Dlv622 and Seu621 exhibited similar efficacy on strain B536-17-2 as on their original host strain (EOP = 1), but showed 100-fold lower efficacy on strain 9faiz (EOP = 0.01). On strain G4-17, the EOP values for Dlv622 and Seu621 were 0.1 and 0.001, respectively. Phage FRZ284 demonstrated lytic activity across all three strains. However, when titrated by Appelman’s method, phages did not completely inhibit bacterial growth even at the highest concentration (10^9^ PFU/mL).

The recombinant depolymerase Dep622, obtained in a previous study (Gorodnichev et al., 2021a), formed halos on all three *K. pneumoniae* strains. The MHF values of Dep622 varied across strains, ranging from 0.68 to 1.37 μg/mL.

### 3.2 Individual and combined effects of antimicrobial agents on planktonic bacterial cells

Sublethal concentrations of phages (10^9^ PFU/well) and maximum concentration of antibiotic in serum (Table 2) were used to study the effects of antimicrobial agents. Depolymerase Dep622 was added at a concentration equivalent to 100 MHF for each strain.

**Figure 1:**
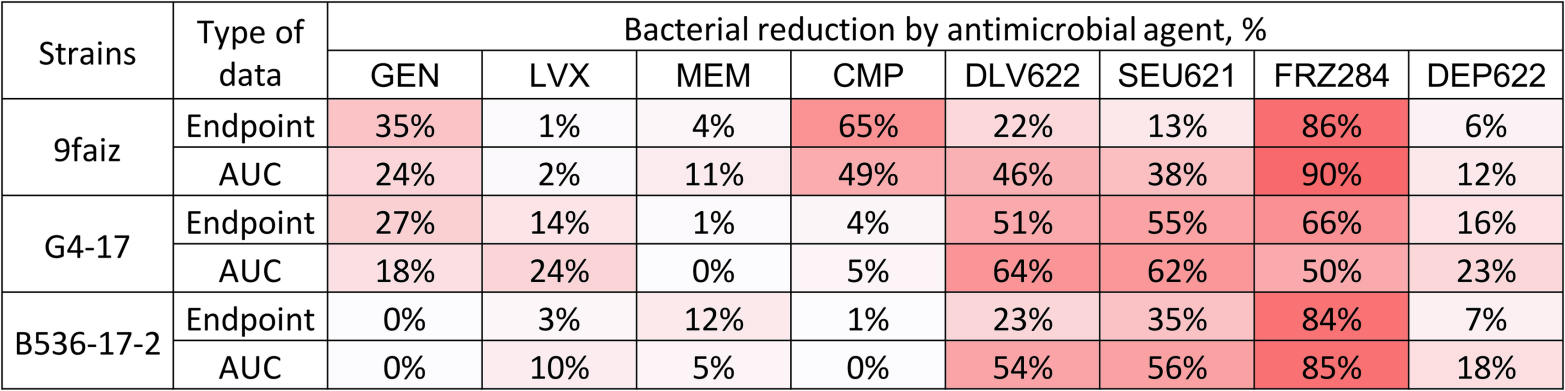
Individual effects (endpoint and AUC) of antibiotics, bacteriophages, and depolymerase on planktonic cells of *K. pneumoniae*. The color gradient shows the degree of reduction. GEN, Gentamicin; LVX, Levofloxacin; MEM, Meropenem; CMP, Chloramphenicol.

The individual application of antibiotics had minimal antimicrobial effect, except for CMP on strain 9faiz (Figure 1). Phages exhibited a more substantial effect: phages Dlv622 and Seu621 reduced endpoint OD_600_ and AUC values by 13–55% and 38–64%, respectively, while phage FRZ284 achieved reductions of up to 90%. Depolymerase treatment reduced endpoint OD_600_ and AUC by no more than 25%.

**Figure 2:**
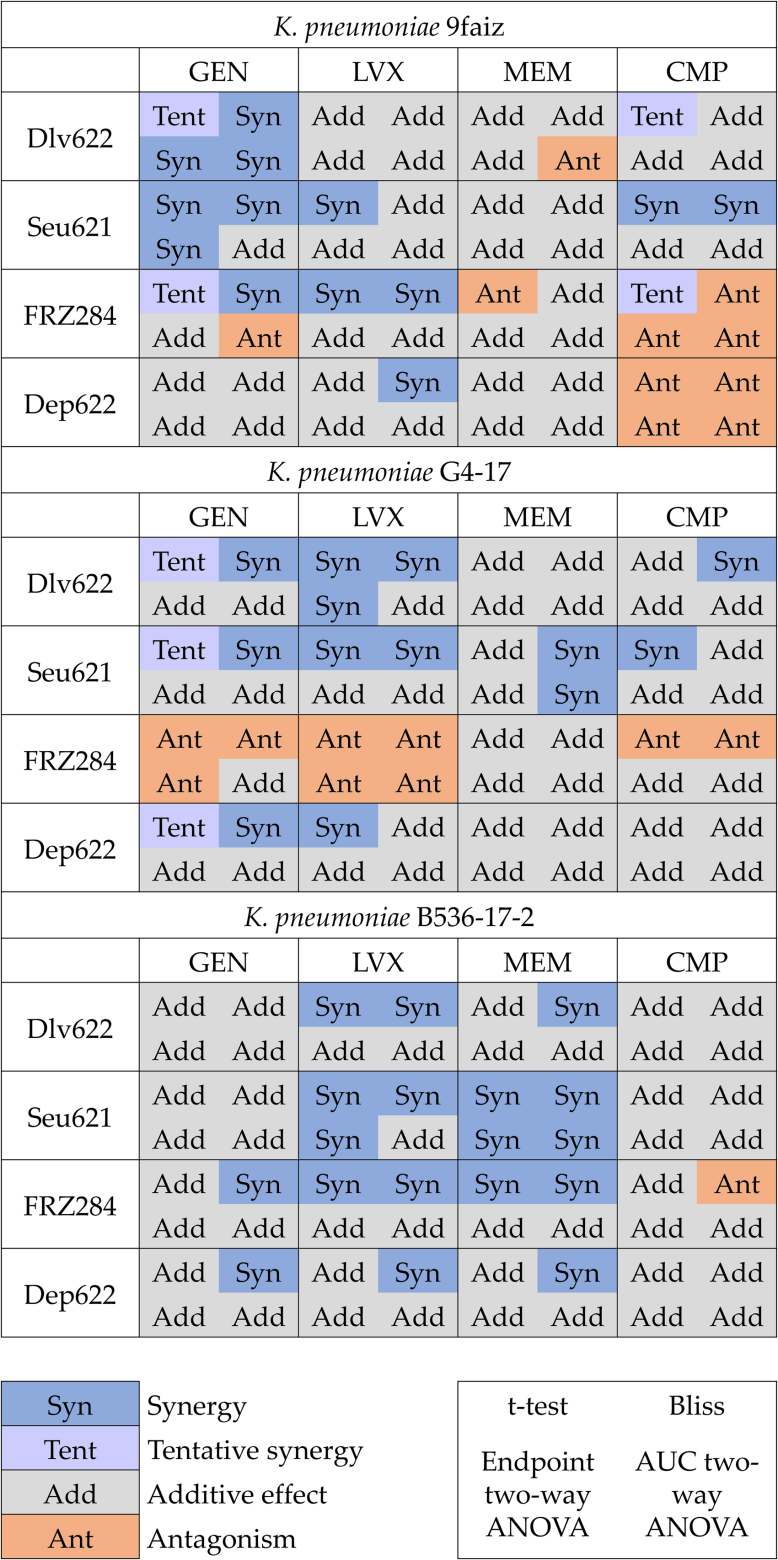
Cases of synergy, tentative synergy, additive effect and antagonism predicted by different methods. *p* < 0.05.

Combined effects were assessed using *t*-test, Bliss, and two-way ANOVA methods, with ANOVA applied to both endpoint and AUC data (Figure 2; Supplementary Figure S1). The *t*-test identified 25 significant cases of phage-antibiotic effects: 13 cases of synergy, 7 cases of tentative synergy, and 5 cases of antagonism. The Bliss method detected 23 synergistic and 6 antagonistic interactions. Two-way ANOVA identified the fewest significant effects: 9 and 8 cases for endpoint OD_600_ and AUC, respectively, with 5 cases of synergy based on endpoint OD_600_ and 3 based on AUC.

### 3.3 Comparison of methods

The *t*-test and Bliss methods demonstrated the highest convergence and number of statistically significant results. Even when statistical significance was not reached by one method, the effect direction (*S*-value magnitude or OD value for the agent combination relative to individual effects) remained consistent across approaches (Figure 2). The term “tentative synergy,” introduced for the *t*-test, showed a strong correlation with Bliss results: of the seven cases of tentative synergy, five aligned with Bliss-defined synergy, one displayed an additive effect, and one showed antagonism (9faiz + FRZ284 + CMP).

Analysis of endpoint OD_600_ data by two-way ANOVA revealed nine effects consistent with those identified by the *t*-test and Bliss. Using AUC data, two-way ANOVA identified eight effects: five combinations (two cases of synergy and three of antagonism) aligned with results based on endpoint OD600, while the remaining three cases (one synergy and two antagonism) indicated a more complex nature of phage-antibiotic interactions. In the case of synergy (G4-17 + Seu621 + MEM), the result was consistent with Bliss but showed no significant difference from the most potent agent alone by *t*-test. In the first antagonism case (9faiz + Dlv622 + MEM), only two-way ANOVA based on AUC values showed a significant effect, whereas in the second (9faiz + FRZ284 + GEN), two-way ANOVA indicated antagonism, contrasting with Bliss-defined synergy and tentative synergy identified by the *t*-test.

Discrepancies in effects identified by different methods (9faiz + FRZ284 + CMP and 9faiz + FRZ284 + GEN) may be explained by the fact that in these cases both antimicrobial agents reduced optical density and the combined effect of their individual actions exceeded 100%. Consequently, synergy in these combinations would only be observed if the combined optical density were negative—a technically unachievable condition. Thus, accurately determining the effect of these combinations is challenging at the phage and antibiotic concentrations used.

### 3.4 Checkerboard assay

A checkerboard assay was conducted for three phage-antibiotic combinations against *K. pneumoniae* strains. For the combination G4-17 + Dlv622 + LVX, synergy was confirmed by all three analytical methods, while for G4-17 + Seu621 + LVX and B536-17-2 + Dlv622 + LVX, synergy was confirmed by both the *t*-test and Bliss analysis (Figure 2).

**Figure 3:**
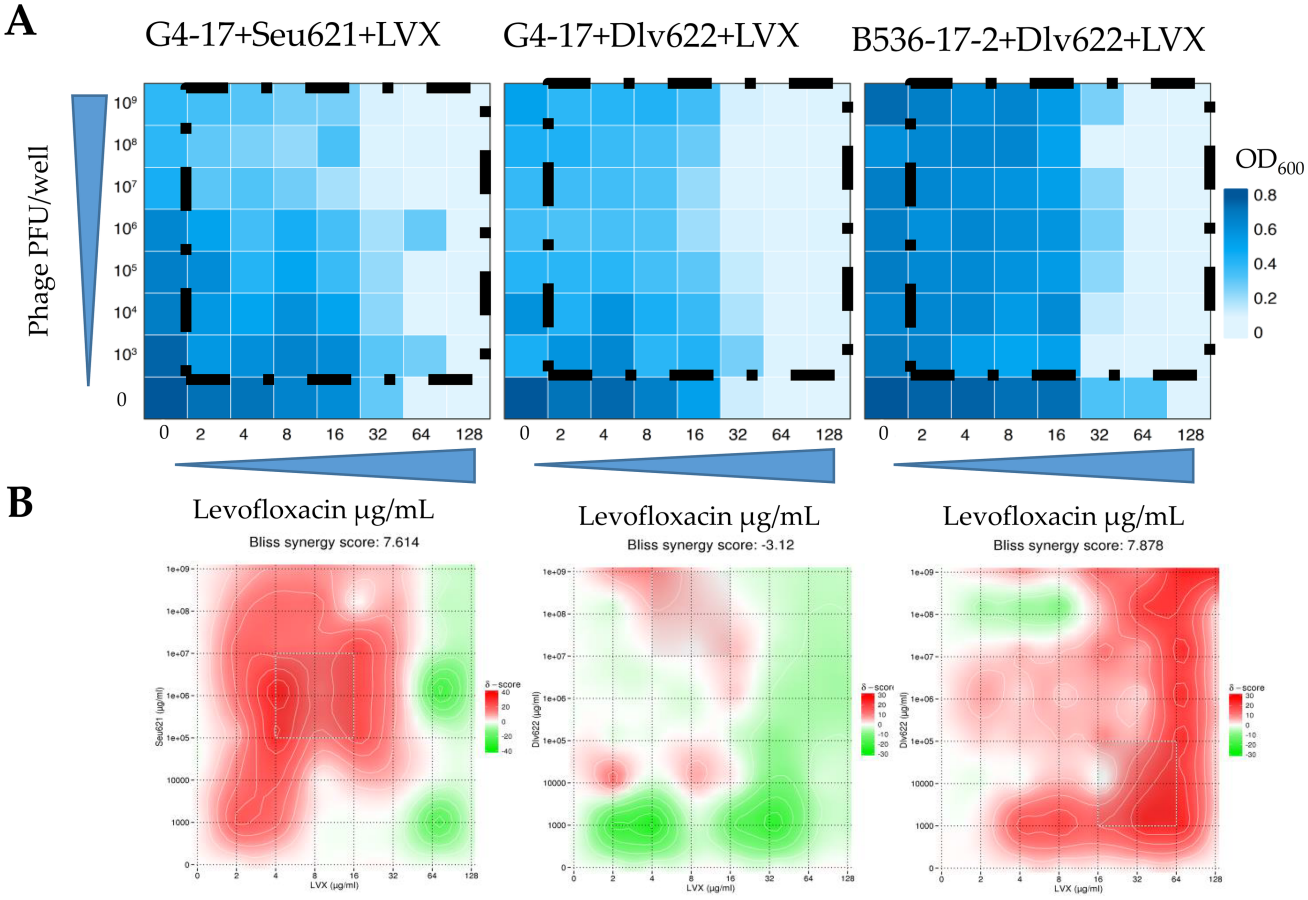
**(A)** Heat plots OD_600_ of checkerboard assays (the color key shows the optical density of the culture at the time of 20 h incubation), **(B)** checkerboard assays analyzed by the Bliss algorithm. Synergy is shown in red, antagonism in green.

The FICI values for G4-17 + Seu621 + LVX and G4-17 + Dlv622 + LVX were 0.5, indicating an additive effect. In contrast, the B536-17-2 + Dlv622 + LVX combination displayed a synergistic effect with a FICI of 0.25 (Figure 3A).

Analysis of these data with Bliss scoring in SynergyFinder v.3.0 showed that the G4-17 + Seu621 + LVX combination exhibited strong synergy at most phage and antibiotic concentrations (Figure 3B). Conversely, G4-17 + Dlv622 + LVX showed weak synergy, with additive and antagonistic effects predominating; however, at concentrations used in previous experiments, Bliss analysis still indicated synergy. For the final combination, B536-17-2 + Dlv622 + LVX, synergy was primarily observed, though antagonism emerged at a phage concentration of 10^8^ PFU/well and antibiotic levels of 4–8 μg/mL, comparable to previous experimental conditions.

## 4 Discussion

In recent years, the scientific community has actively explored the potential effects of combined phage-antibiotic therapy. Numerous *in vitro* studies have demonstrated both synergistic and antagonistic interactions (Schindler, 1964; Tagliaferri et al., 2019; Jiang et al., 2020; Liu et al., 2020; Nicholls et al., 2023; Shein et al., 2024), and these effects have been further validated in *in vivo* models (Oechslin et al., 2017; Grygorcewicz et al., 2020; Santamaría-Corral et al., 2023). These findings highlight the potential for translating such combined approaches into clinical practice, offering promising avenues for improved therapeutic outcomes.

One of the key challenges in studying phage-antibiotic combinations is the difficulty in comparing results across studies due to significant differences in experimental methods, limited characterization of bacterial strains and phages, and a restricted number of tested combinations. These limitations often result in a tendency to describe synergistic effects as unique to specific phage-antibiotic pairs (Bedi et al., 2009; Xu et al., 2022; Zhao et al., 2023). In our study, we aimed to carefully standardize and structure each phase of evaluating the combined effects of phages and antibiotics on *K. pneumoniae*.

In selecting *K. pneumoniae* strains, we focused on isolates that were likely to require combination therapy for eradication. We included multidrug-resistant strains with reduced susceptibility to phages, specifically with capsular type KL23 and clinically significant sequence types ST39 and ST1869 (Table 2). The KL23 capsular type is found in approximately 4% of the general population but accounts for 9–17% of carbapenemase-producing strains (Satlin et al., 2017; Sonda et al., 2018). ST39 is associated with several outbreaks of carbapenem-resistant *K. pneumoniae* in Russia and Greece (Kuzina et al., 2023; Tryfinopoulou et al., 2023). ST1869, although rare in clinical practice, exhibits carbapenem resistance (Li et al., 2019) and differs by only one allele from ST11, a prevalent sequence type in China known for high rates of multidrug resistance and hypervirulence (Gu et al., 2018).

To maximize the diversity of observed effects, we selected phages from different taxonomic families: Dlv622 (*Autographiviridae*, *Slopekvirinae*, *Drulisvirus*), Seu621 (*Vequintavirinae*, *Mydovirus*), and FRZ284 (*Straboviridae*, *Tevenvirinae*, *Jiaodavirus*). The lytic phage FRZ284, capable only of lysis from without, was included to demonstrate that even phages unlikely to be chosen for therapy based on spot tests can exhibit synergy with antibiotics. This phage lysed *K. pneumoniae* strains regardless of capsular type and has a receptor-binding protein of unknown specificity (Gorodnichev et al., 2021b).

The phages Dlv622 and Seu621 possess homologous receptor-binding proteins (query coverage 98%, identity 65.94%), characterized as polysaccharide depolymerases (Gorodnichev et al., 2021a). One of these proteins, Dep622, the receptor-binding protein of phage Dlv622, was used in the current study. Since depolymerase itself does not lyse bacterial cells, investigating patterns in the combined effects of depolymerases and antibiotics offers a novel perspective on the therapeutic application of depolymerases.

We also selected antibiotics from different classes (aminoglycosides, fluoroquinolones, carbapenems, and phenicols) to maximize the diversity of observed effects. These antibiotics are widely used in clinical practice: meropenem is generally considered a first-line treatment for serious *K. pneumoniae* infections, while gentamicin is frequently used as part of combination therapy for severe infections. The use of chloramphenicol and levofloxacin is typically guided by susceptibility testing results to ensure their effectiveness against resistant strains (CLSI, 2021).

The concentrations of antimicrobial agents used in phage-antibiotic combination studies are pivotal for interpreting results and assessing clinical relevance. Most studies on phage-antibiotic interactions employ sublethal doses of antimicrobials, which may be above or below therapeutic doses depending on the bacterial strain’s susceptibility. In this study, we based antibiotic concentrations on C_max_—the maximum serum concentration of an antibiotic reached after a single dose (Table 1). We believe this approach enhances clinical relevance, providing a more clinically relevant evaluation of combination efficacy. While achieving an equivalent concentration for phages would be ideal, this is likely unfeasible due to phage replication at infection sites.

To assess and compare combined antimicrobial effects, we used several established methods: the *t*-test, Bliss independence model, two-way ANOVA, and checkerboard assay (Liu et al., 2020; Eller et al., 2021; Gordillo Altamirano et al., 2022; Nikolic et al., 2022; Xu et al., 2022). Among these, the *t*-test, commonly applied in many studies, only compares the combined effect of agents with the effect of the most effective agent alone. This approach does not account for the contribution of the second agent, thus limiting its ability to determine the overall interaction effect. To address this limitation, we refined the criteria for *t*-test interpretation, defining conditions for synergy, antagonism, and additive effects. In specific cases where the individual effects of both agents were statistically significantly different from the control and the inhibitory effect of their combination was significantly stronger, we introduced the term “tentative synergy.” This term reflects a potential positive interaction, yet the exact contribution of the second agent remains uncertain, precluding a definitive classification as synergy.

Another frequently used method is the Bliss independence model, which assumes that two agents act independently through stochastic processes (Eller et al., 2021). Given that antibiotics and bacteriophages target bacteria via distinct biochemical mechanisms, the Bliss model is applicable for analyzing their combined effects. However, a notable drawback of this method is its high sensitivity—it does not account for the absence of an effect. Consequently, if the effects of both agents and their combination do not significantly differ from the control, the Bliss model will classify the interaction as additive, even when a true interaction might be absent.

Another commonly used approach for assessing interactions is two-way ANOVA, which specifically evaluates the interaction term, making it relevant for distinguishing between synergy and antagonism. However, the interaction *p*-value alone does not provide information on the direction of the effect. This interpretation can be inferred from the slope of interaction plots (Liu et al., 2020), though standard error may limit the accuracy of this approach.

Our results indicate that the *t*-test and Bliss models identified the highest number of statistically significant effects, likely due to the lack of adjustments for multiple comparisons, allowing for the detection of less pronounced synergy and antagonism effects (Figure 2). In contrast, the more conservative two-way ANOVA, which requires a strong and consistent effect to reach statistical significance, identified considerably fewer effects.

The broad range of results highlights the need to select methods based on specific research objectives. For selecting potential phage-antibiotic combinations for therapeutic application, using the *t*-test or Bliss (or a combination of these) may be optimal due to their simplicity in calculation and interpretation. Additionally, for therapeutic purposes, identifying potential antagonistic interactions can be more critical than seeking combinations with the highest synergy. In turn, two-way ANOVA could be an effective tool for exploratory research, particularly when the objective is to pinpoint combinations with strong interactions for deeper investigation into bacterial response.

The checkerboard assay, with its evaluation of interactions across a broad concentration range, has unique advantages. While this approach appears more informative, the FICI values calculated for two out of three cases conflicted with the effects observed in *t*-test, Bliss, and two-way ANOVA analyses. Notably, the checkerboard assay operates at antimicrobial agent concentrations unlikely to be achieved in real therapeutic scenarios. However, checkerboard results, when supplemented with Bliss analysis, can illustrate how the combined effect varies with agent concentrations. For example, when using linezolid near C_max_ (4–8 μg/mL), the combinations G4-17 + Seu621 + LVX and B536-2-17 + Dlv622 + LVX consistently demonstrate synergy at any phage concentration, while a similar effect for G4-17 + Dlv622 + LVX is observed only at the highest phage concentrations. Such observations may be valuable for understanding the potential variability in phage titers at infection sites (Schooley et al., 2017).

Overall, while these methods cannot be directly compared due to differences in statistical assumptions and sensitivity, their complementary use provides a more robust assessment of phage-antibiotic interactions. The consistency of results across multiple methods strengthens confidence in observed effects, whereas discrepancies highlight potential context-dependent factors such as dose–response relationships. These findings emphasize the importance of selecting the appropriate analytical framework based on the specific goals of a study rather than relying on a single method.

**Figure 4:**
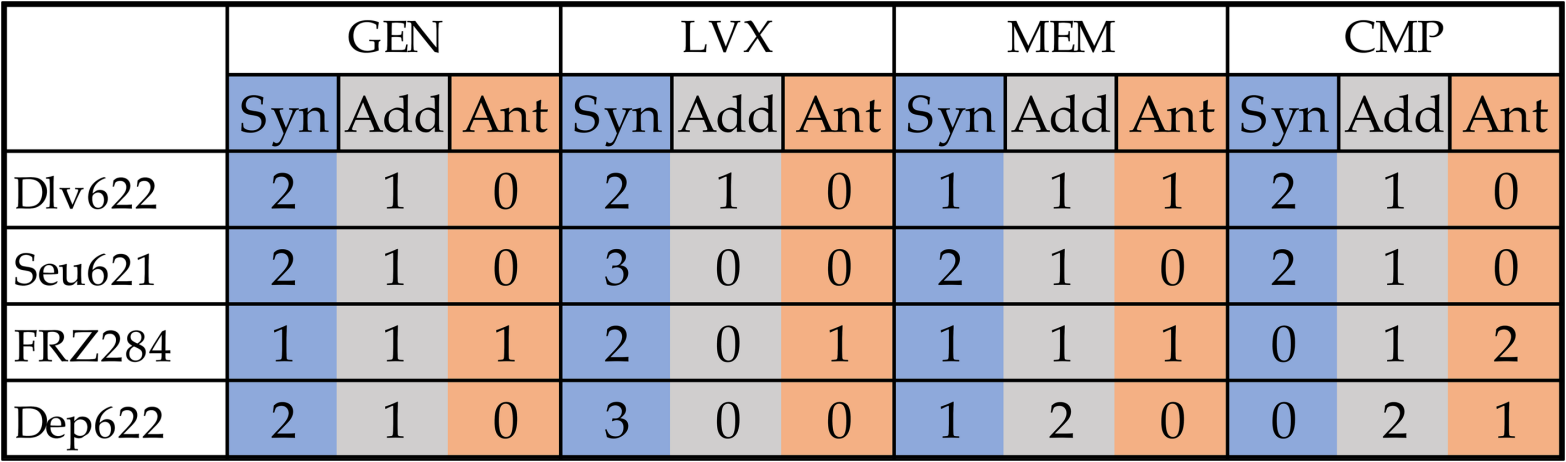
Cumulative effects of combinations of antibiotics and phages or depolymerase on all three strains from a practical point of view. Synergy is shown in blue, antagonism in orange, additive effect in gray.

In total, 35 of the 48 phage-antibiotic combinations tested demonstrated statistically significant effects based on at least one method (Figure 4). Two previously described cases (9faiz + FRZ284 + CMP and 9faiz + FRZ284 + GEN) were excluded due to difficulties in accurately interpreting their effects; these combinations were thus classified as statistically insignificant.

Phage and depolymerase combinations with levofloxacin exhibited the highest proportion of synergistic effects. However, only the combinations of levofloxacin with Seu621 phage or Dep622 depolymerase consistently produced effects across all three *K. pneumoniae* strains tested. Previous studies have shown synergy between fluoroquinolone antibiotics and phages in disrupting *K. pneumoniae* biofilms, specifically with a ciprofloxacin and T7-like phage combination (Verma et al., 2009); however, this is the first report of synergy involving a *Mydovirus* phage or depolymerase. Nevertheless, expanding the diversity of bacterial strains tested may impact the consistent synergistic interaction observed between levofloxacin and either Seu621 phage or Dep622 depolymerase. In fact, as shown in the checkerboard assay, certain effects may reverse depending on the antimicrobial concentration (Figure 3).

Gentamicin ranked second in terms of the proportion of synergistic effects observed. Although synergistic effects between aminoglycoside antibiotics and *K. pneumoniae* phages have been reported in animal models (Wang et al., 2021; Zhao et al., 2023), numerous studies suggest that aminoglycosides may inhibit phages (Schindler, 1964; Bowman, 1967; Jiang et al., 2020; Kever et al., 2022). In our study, all phage and depolymerase combinations with gentamicin showed trends toward synergy or additive effects, with antagonism detected in only one case.

Phage combinations with meropenem and chloramphenicol yielded the highest proportion of statistically insignificant results and a higher frequency of antagonistic effects. Beta-lactam antibiotics, particularly meropenem, are frequently explored in studies examining phage synergy. For example, meropenem combined with a *K. pneumoniae Webervirus* phage showed synergy in biofilm degradation (Xu et al., 2022), while amoxicillin reduced biofilm biomass when combined with an unclassified phage (Bedi et al., 2009). Synergy between *K. pneumoniae* phages and chloramphenicol has not been previously documented; however, similar effects have been observed with a *P. aeruginosa* N4-like phage KPP21 (Uchiyama et al., 2018) and *Inovirus* phages (Hagens et al., 2006).

Notably, combinations involving the FRZ284 phage and the Dep622 depolymerase revealed diverse effects. In plating assays, phage FRZ284 only displayed lysis from without, indicating that while it could attach to bacteria, it could not proliferate within the strain. Despite this, cases of synergy were observed with three out of four antibiotics (gentamicin, levofloxacin, and meropenem), alongside antagonistic effects with all four antibiotics. The Dep622 depolymerase itself did not directly kill bacteria but sensitized them to antimicrobial agents. In this context, synergistic effects with antibiotics were expected, as demonstrated with gentamicin, levofloxacin, and meropenem. However, the antagonistic effect observed with chloramphenicol on strain 9faiz—validated across all testing methods—remains challenging to interpret.

Combinations of phages Dlv622 or FRZ284 with meropenem, as well as FRZ284 with gentamicin, demonstrated all three types of interactions (synergy, additive effect, and antagonism) across the three *K. pneumoniae* strains. This unpredictability stems not only from methodological inconsistencies but also from the genetic diversity of bacterial strains and the variability of phage lifecycles, which significantly influence outcomes.

The numerous studies have described the mechanisms underlying synergy and antagonism in phage-antibiotic interactions (Liu et al., 2020; Zuo et al., 2021; Bulssico et al., 2023; Luo et al., 2023; Pons et al., 2023; Kunz Coyne et al., 2024; Qin et al., 2024; Yu et al., 2025). However, it is important to recognize that these mechanisms have been described for specific bacteria-phage-antibiotic combinations, and given the vast diversity of bacterial strains and phages, they may not represent the only possible explanations. The variability in effects observed in our study further supports the idea that multiple, context-dependent factors influence phage-antibiotic interactions. Given this complexity, predicting the clinical applicability of specific phage-antibiotic combinations requires careful experimental validation, but their translation into clinical settings remains uncertain without standardized experimental models.

To advance the field, standardizing experimental approaches for assessing combination effects is crucial. The goal of our study is to initiate this discussion by demonstrating how methodological variability can lead to inconsistent interpretations.

Ultimately, the development of effective phage-antibiotic therapies requires a shift from studying isolated mechanisms to a systematic, standardized framework that accounts for the complexity of microbial interactions. Establishing such approaches will improve reproducibility and enable the rational design of phage-based treatments in clinical practice.

## 5 Conclusion

The combined application of phages and antibiotics in therapy requires reliable methods to assess their interactions; however, the lack of standardized approaches complicates data interpretation and clinical implementation. In this study, we applied the most commonly used methods (*t*-test, Bliss method, two-way ANOVA, and checkerboard assay), complemented by the standardization of antibiotic concentrations at C_max_. The *t*-test proved valuable for rapid interaction assessment, especially after expanding the criteria and introducing “tentative synergy,” allowing consideration of complex cases where the contribution of the second agent is less apparent. The Bliss method aligned well with the *t*-test results and was particularly effective when combined with checkerboard assay data, enabling visualization of the interaction effect based on agent concentration. In contrast, the FICI was less informative and poorly correlated with other methods. Two-way ANOVA identified only the most consistent and substantial effects, highlighting strong interactions and making it useful for comprehensive investigations.

Overall, our approach identified only two combinations with consistent effects across all three *K. pneumoniae* strains, suggesting that synergy or antagonism is not solely determined by the nature of phage or antibiotic but depends on a complex interplay of factors. These findings emphasize the need for standardized interaction assessment methods to improve comparability of data and enhance the efficacy of combination therapies.

## Data Availability

The original contributions presented in the study are included in the article/Supplementary material, further inquiries can be directed to the corresponding author.
