# Supplementary material for: Phage-antibiotic combinations against Klebsiella pneumoniae: impact of methodological approaches on effect evaluation
Source: Front Microbiol. 2025 Mar 12;16:1530819. doi: 10.3389/fmicb.2025.1530819 (PMC11937024; doi:10.3389/fmicb.2025.1530819)
Supplement: Supplementary file 1 [file Data_Sheet_1.pdf]

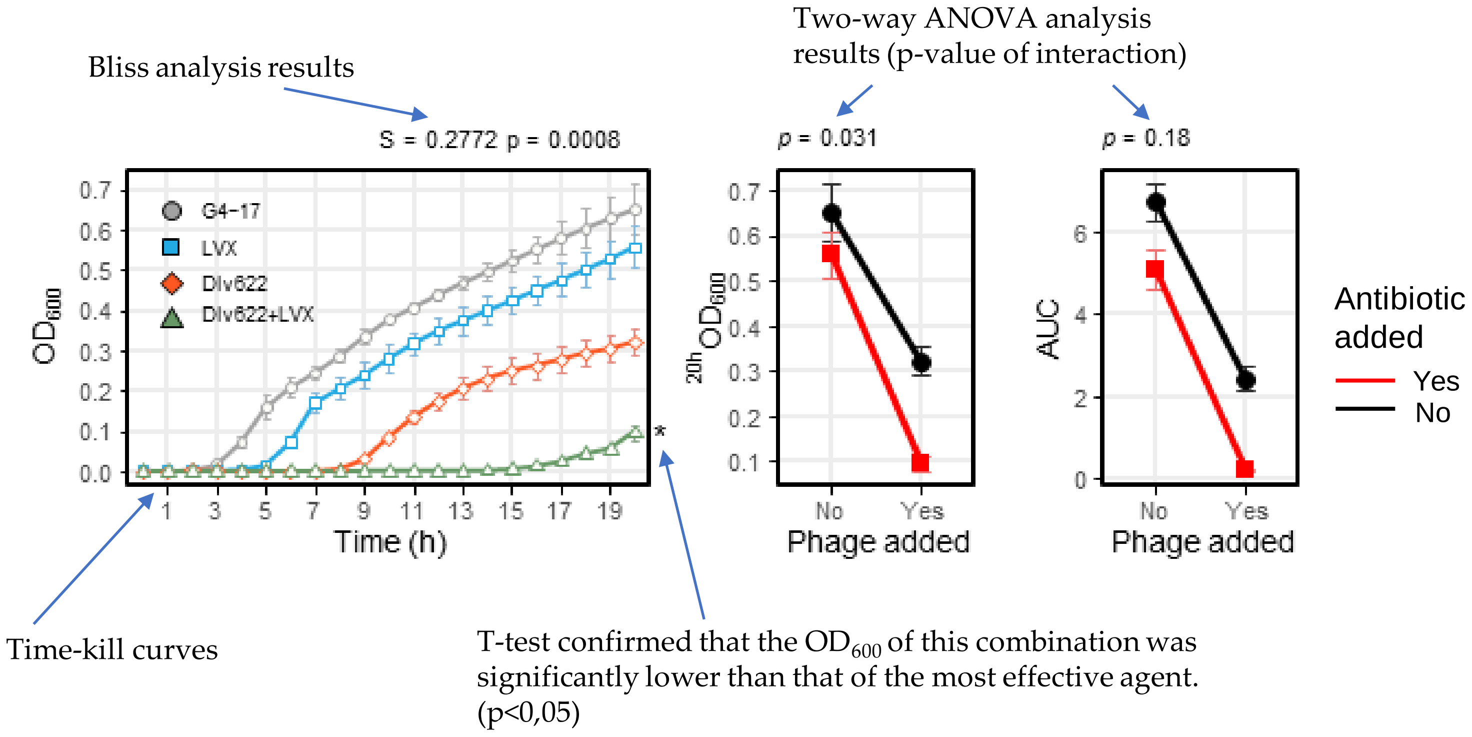

Supplementary Figure S1. Outcomes of individual and combined effects of antibiotics and bacteriophages on *K. pneumoniae* strains

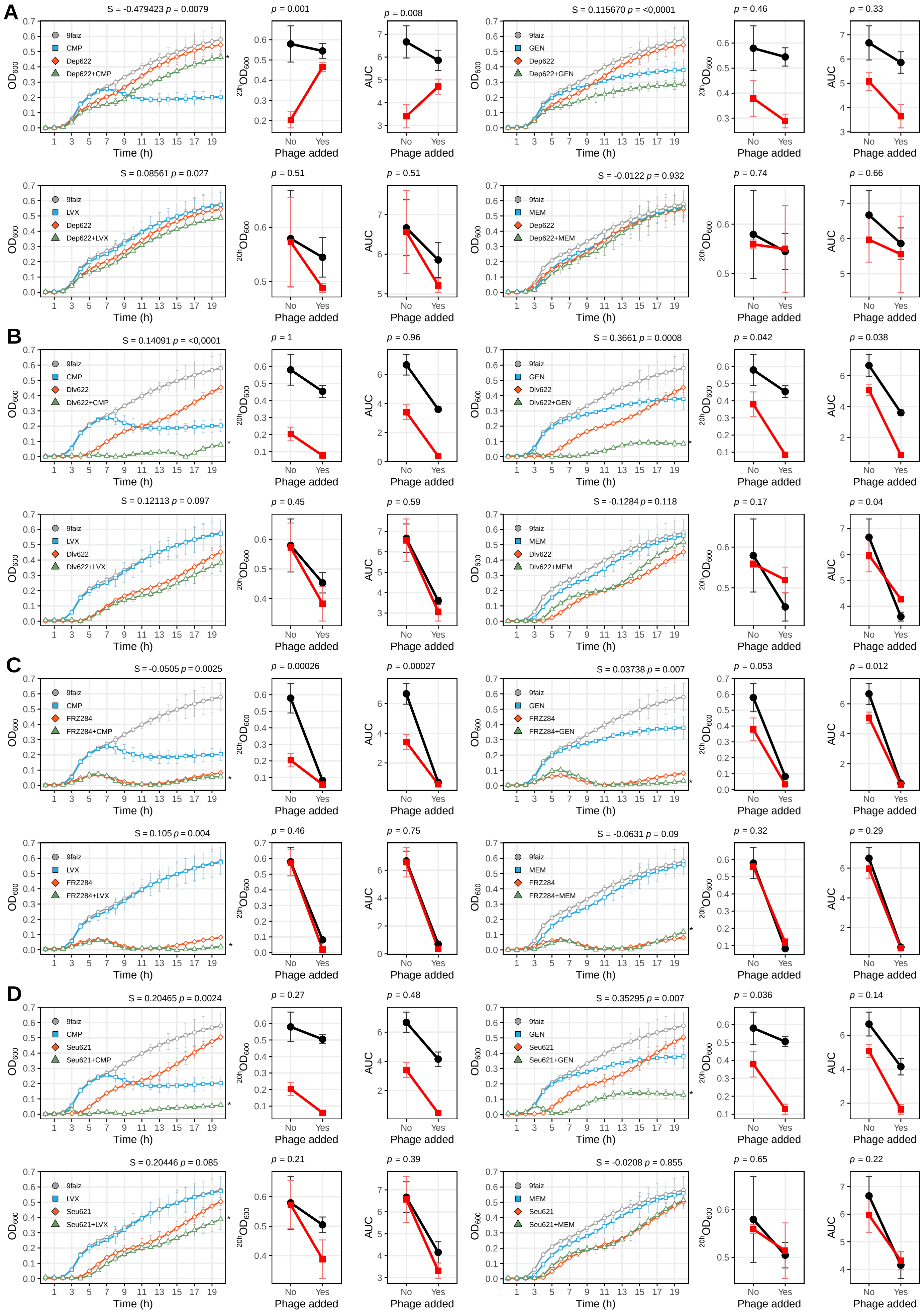
A
S = -0.479423 p = 0.0079
9faiz
CMP
Dep622
Dep622+CMP
OD600
Time (h)
p = 0.001
20hOD600
Phage added
No
Yes
p = 0.008
AUC
S = 0.115670 p = <0,0001
GEN
Dep622+GEN
p = 0.46
p = 0.33
S = 0.08561 p = 0.027
LVX
Dep622+LVX
p = 0.51
p = 0.51
S = -0.0122 p = 0.932
MEM
Dep622+MEM
p = 0.74
p = 0.66
B
S = 0.14091 p = <0,0001
Dlv622
Dlv622+CMP
p = 1
p = 0.96
S = 0.3661 p = 0.0008
Dlv622+GEN
p = 0.042
p = 0.038
S = 0.12113 p = 0.097
Dlv622+LVX
p = 0.45
p = 0.59
S = -0.1284 p = 0.118
Dlv622+MEM
p = 0.17
p = 0.04
C
S = -0.0505 p = 0.0025
FRZ284
FRZ284+CMP
p = 0.00026
p = 0.00027
S = 0.03738 p = 0.007
FRZ284+GEN
p = 0.053
p = 0.012
S = 0.105 p = 0.004
FRZ284+LVX
p = 0.46
p = 0.75
S = -0.0631 p = 0.09
FRZ284+MEM
p = 0.32
p = 0.29
D
S = 0.20465 p = 0.0024
Seu621
Seu621+CMP
p = 0.27
p = 0.48
S = 0.35295 p = 0.007
Seu621+GEN
p = 0.036
p = 0.14
S = 0.20446 p = 0.085
Seu621+LVX
p = 0.21
p = 0.39
S = -0.0208 p = 0.855
Seu621+MEM
p = 0.65
p = 0.22

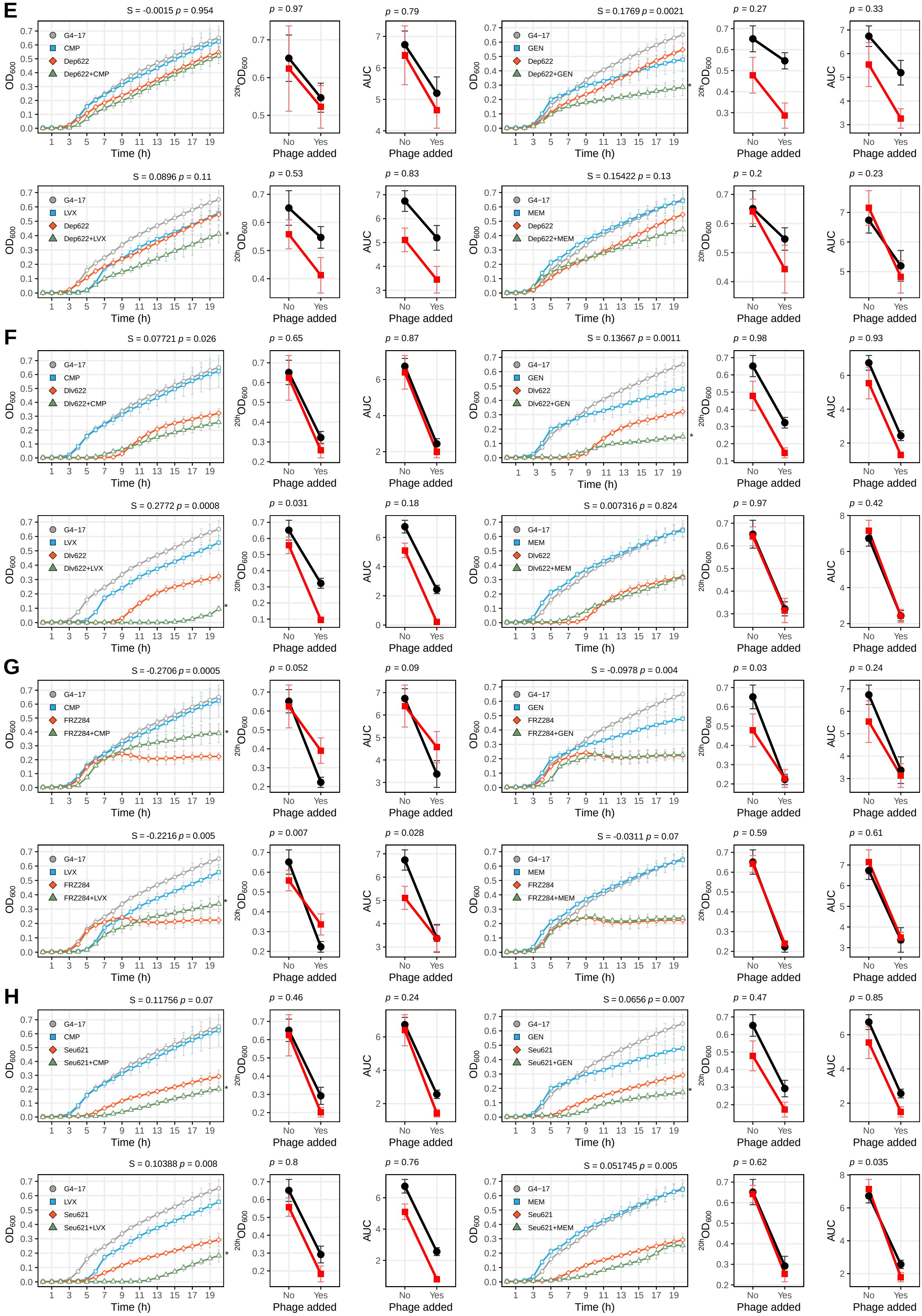
E
S = -0.0015 p = 0.954
G4−17
CMP
Dep622
Dep622+CMP
OD600
Time (h)
p = 0.97
20hOD600
No
Yes
Phage added
p = 0.79
AUC
S = 0.1769 p = 0.0021
G4−17
GEN
Dep622
Dep622+GEN
p = 0.27
p = 0.33
S = 0.0896 p = 0.11
G4−17
LVX
Dep622
Dep622+LVX
p = 0.53
p = 0.83
S = 0.15422 p = 0.13
G4−17
MEM
Dep622
Dep622+MEM
p = 0.2
p = 0.23
F
S = 0.07721 p = 0.026
G4−17
CMP
Dlv622
Dlv622+CMP
p = 0.65
p = 0.87
S = 0.13667 p = 0.0011
G4−17
GEN
Dlv622
Dlv622+GEN
p = 0.98
p = 0.93
S = 0.2772 p = 0.0008
G4−17
LVX
Dlv622
Dlv622+LVX
p = 0.031
p = 0.18
S = 0.007316 p = 0.824
G4−17
MEM
Dlv622
Dlv622+MEM
p = 0.97
p = 0.42
G
S = -0.2706 p = 0.0005
G4−17
CMP
FRZ284
FRZ284+CMP
p = 0.052
p = 0.09
S = -0.0978 p = 0.004
G4−17
GEN
FRZ284
FRZ284+GEN
p = 0.03
p = 0.24
S = -0.2216 p = 0.005
G4−17
LVX
FRZ284
FRZ284+LVX
p = 0.007
p = 0.028
S = -0.0311 p = 0.07
G4−17
MEM
FRZ284
FRZ284+MEM
p = 0.59
p = 0.61
H
S = 0.11756 p = 0.07
G4−17
CMP
Seu621
Seu621+CMP
p = 0.46
p = 0.24
S = 0.0656 p = 0.007
G4−17
GEN
Seu621
Seu621+GEN
p = 0.47
p = 0.85
S = 0.10388 p = 0.008
G4−17
LVX
Seu621
Seu621+LVX
p = 0.8
p = 0.76
S = 0.051745 p = 0.005
G4−17
MEM
Seu621
Seu621+MEM
p = 0.62
p = 0.035

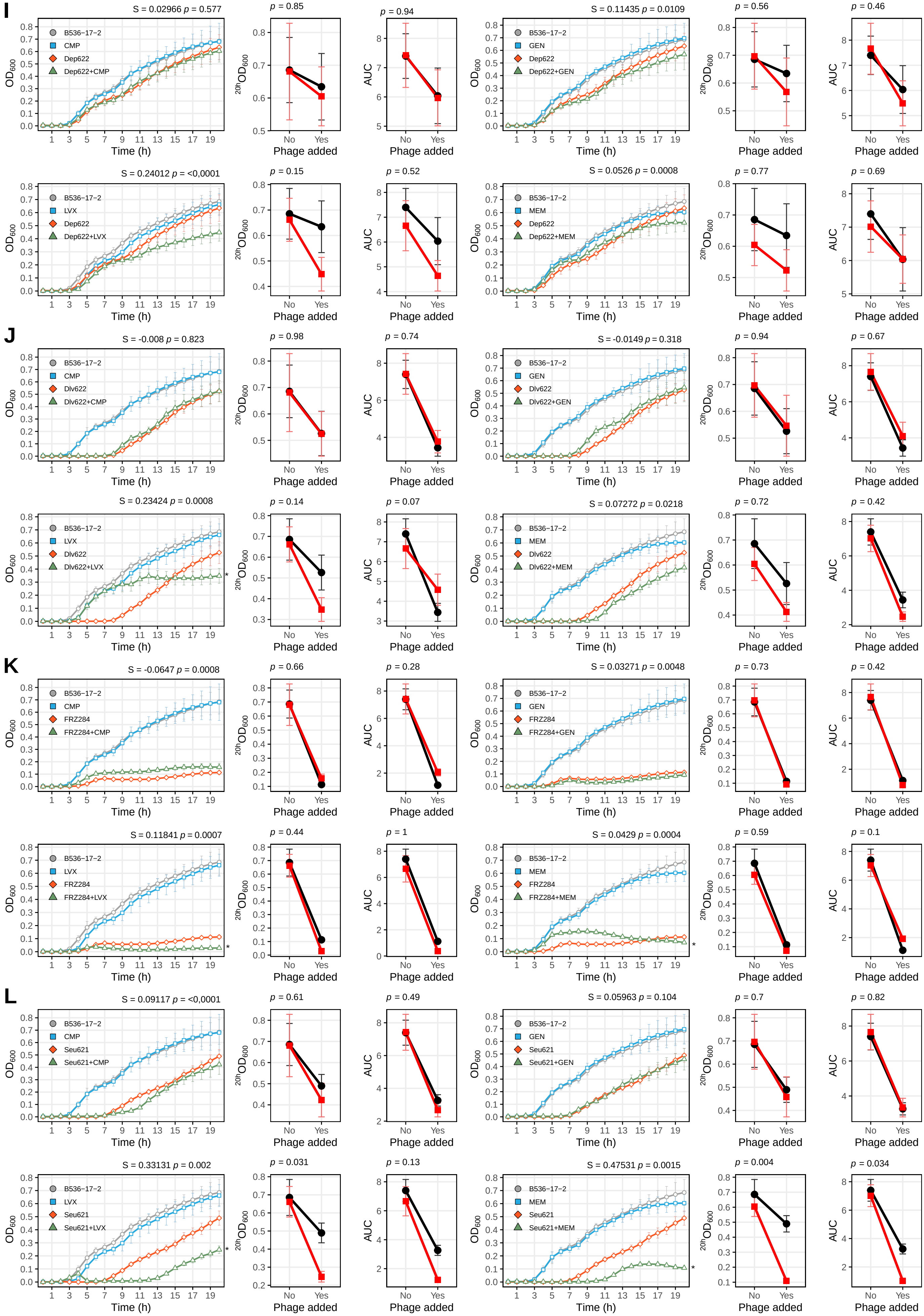
I
S = 0.02966 p = 0.577
B536–17–2
CMP
Dep622
Dep622+CMP
OD600
Time (h)
p = 0.85
20hOD600
Phage added
No
Yes
p = 0.94
AUC
S = 0.11435 p = 0.0109
GEN
Dep622+GEN
p = 0.56
p = 0.46
S = 0.24012 p = <0,0001
LVX
Dep622+LVX
p = 0.15
p = 0.52
S = 0.0526 p = 0.0008
MEM
Dep622+MEM
p = 0.77
p = 0.69
J
S = -0.008 p = 0.823
Dlv622
Dlv622+CMP
p = 0.98
p = 0.74
S = -0.0149 p = 0.318
Dlv622+GEN
p = 0.94
p = 0.67
S = 0.23424 p = 0.0008
Dlv622+LVX
p = 0.14
p = 0.07
S = 0.07272 p = 0.0218
Dlv622+MEM
p = 0.72
p = 0.42
K
S = -0.0647 p = 0.0008
FRZ284
FRZ284+CMP
p = 0.66
p = 0.28
S = 0.03271 p = 0.0048
FRZ284+GEN
p = 0.73
p = 0.42
S = 0.11841 p = 0.0007
FRZ284+LVX
p = 0.44
p = 1
S = 0.0429 p = 0.0004
FRZ284+MEM
p = 0.59
p = 0.1
L
S = 0.09117 p = <0,0001
Seu621
Seu621+CMP
p = 0.61
p = 0.49
S = 0.05963 p = 0.104
Seu621+GEN
p = 0.7
p = 0.82
S = 0.33131 p = 0.002
Seu621+LVX
p = 0.031
p = 0.13
S = 0.47531 p = 0.0015
Seu621+MEM
p = 0.004
p = 0.034
